# Efficacy and Safety of Cyclosporine in Acute Myocardial Infarction: A Systematic Review and Meta-Analysis

**DOI:** 10.3389/fphar.2018.00238

**Published:** 2018-06-19

**Authors:** Firdaus A. Rahman, Siti S. Abdullah, Wan Zanariah W. A. Manan, Loh Teng-Hern Tan, Chin-Fen Neoh, Long Chiau Ming, Kok-Gan Chan, Learn-Han Lee, Bey-Hing Goh, Shahrzad Salmasi, David Bin-Chia Wu, Tahir M. Khan

**Affiliations:** ^1^Faculty of Pharmacy, Universiti Teknologi MARA, Puncak Alam, Malaysia; ^2^Novel Bacteria and Drug Discovery Research Group, School of Pharmacy, Monash University, Bandar Sunway, Malaysia; ^3^School of Pharmacy, KPJ Healthcare University College, Nilai, Malaysia; ^4^International Genome Centre, Jiangsu University, Zhenjiang, China; ^5^Division of Genetics and Molecular Biology, Faculty of Science, Institute of Biological Sciences, University of Malaya, Kuala Lumpur, Malaysia; ^6^Center of Health Outcomes Research and Therapeutic Safety (Cohorts), School of Pharmaceutical Sciences, University of Phayao, Phayao, Thailand; ^7^School of Pharmacy, Monash University, Bandar Sunway, Malaysia; ^8^Biofunctional Molecule Exploratory Research Group, School of Pharmacy, Monash University, Bandar Sunway, Malaysia; ^9^Asian Centre for Evidence Synthesis in Population, Implementation and Clinical Outcomes, Health and Well-being Cluster, Global Asia in the 21st Century Platform, Monash University, Bandar Sunway, Malaysia; ^10^Collaboration for Outcomes Research and Evaluation, Faculty of Pharmaceutical Sciences, University of British Columbia, Vancouver, BC, Canada; ^11^Institute of Pharmaceutical Science, University of Veterinary and Animal Science, Lahore, Pakistan

**Keywords:** cyclosporine, acute myocardial infraction, meta-analysis, efficacy, safety

## Abstract

There are various studies that have addressed the use of Cyclosporine among patients with acute myocardial infarction (AMI). However, to date there is hardly any concise and systematically structured evidence that debate on the efficacy and safety of Cyclosporine in AMI patients. The aim of this review is to systematically summarize the overall evidence from published trials, and to conduct a meta-analysis in order to determine the efficacy and safety of Cyclosporine vs. placebo or control among patients with AMI. All randomized control trial (RCT) published in English language from January 2000 to August 2017 were included for the systematic review and meta-analysis. A total of six RCTs met the inclusion and were hence included in the systematic review and meta-analysis. Based on the performed meta-analysis, no significant difference was found between Cyclosporine and placebo in terms of left ventricular ejection fraction (LVEF) improvement (mean difference 1.88; 95% CI −0.99 to 4.74; *P* = 0.2), mortality rate (OR 1.01; 95% Cl 0.60 to 1.67, *P* = 0.98) and recurrent MI occurrence (OR 0.65; 95% Cl 0.29 to 1.45, *P* = 0.29), with no evidence of heterogeneity, when given to patients with AMI. Cyclosporine also did not significantly lessen the rate of rehospitalisation in AMI patients when compared to placebo (OR 0.91; 95% Cl 0.58 to 1.42, *P* = 0.68), with moderate heterogeneity (*I*^2^ = 46%). There was also no significant improvement in heart failure events between Cyclosporine and placebo in AMI patients (OR 0.63; 95% Cl 0.31 to 1.29, *P* = 0.21; *I*^2^ = 80%). No serious adverse events were reported in Cyclosporine group across all studies suggesting that Cyclosporine is well tolerated when given to patients with AMI. The use of Cyclosporine in this group of patients, however, did not result in better clinical outcomes vs. placebo at improving LVEF, mortality rate, recurrent MI, rehospitalisation and heart failure event.

## Introduction

Cardiovascular disease poses tremendous burden on public health, as well as the global economy. According to the World Health Organization, an estimated 17.5 million people died from cardiovascular disease in 2012, accounting for 31% mortality globally (WHO, 2016) with an annual cost of $193.1 billion in health-care management and ~$123 billion in productivity loss as a result of premature death (Mozaffarian et al., 2016). Acute myocardial infarction (AMI) is the most severe manifestation of coronary heart disease and has been the leading cause of morbidity and mortality. Myocardial infarction (MI) is defined as myocardial necrosis that is caused by myocardial ischaemia (Thygesen et al., 2012). MI that is associated with incessant electrocardiographic (ECG) ST elevation and subsequent release of biomarkers of myocardial cell death is classified as ST elevation MI (STEMI) (Thygesen et al., 2012; O'Gara et al., 2013) and constitute 25–40% of MI cases, of whom 5% experienced cardiac arrest during hospitalization (O'Gara et al., 2013).

Reperfusion therapy, either by percutaneous coronary intervention (PCI) or intravenous fibrinolysis therapy, remains the mainstay therapeutic management of STEMI (Steg et al., 2012). Primarily, reperfusion therapy is necessary to resuscitate the ischemic or hypoxic myocardium, thereby reducing infarct size and improving left ventricular function. According to both US and European guidelines, reperfusion therapy is recommended to be administrated as quickly and effectively as possible for STEMI (Steg et al., 2012; O'Gara et al., 2013). Primary PCI is the preferable reperfusion therapy for acute STEMI compared to fibrinolytic therapy, especially when there are short time-to-treatment delays and a well-equipped facilities with experienced cardiologists (O'Gara et al., 2013). In many trials, primary PCI has been shown effective in reducing mortality, reinfarction, and stroke due to its high mechanical reperfusion rate (Widimsky et al., 2010; O'Gara et al., 2013). Thus, timely PCI is important to facilitate infarct size reduction in order to optimize myocardial salvage and reduce mortality rate (Ndrepepa, 2015).

Paradoxically, although reperfusion therapy is essential for myocardial salvage, reperfusion of an ischemic area may result in cardiomyocyte dysfunction, a phenomenon termed as reperfusion injury (Verma et al., 2002). The restoration of blood flow during reperfusion therapy triggers the sudden activation of aerobic metabolism and adenosine triphosphate (ATP) production in the presence of calcium overload, which leads to hypercontraction of cardiomyocytes (Ndrepepa, 2015). Reactive oxygen species produced during sudden reactivation of aerobic metabolism induces oxidative stress. The combination of oxidative stress and calcium overload provide ideal conditions for the opening of mitochondrial permeability transition pore (MPTP). The opening of MPTP renders the inner mitochondrial membrane non-selectively permeable to solutes up to 1.5kDa, resulting in the collapse of mitochondrial inner membrane potential, dissociation of oxidative phosphorylation and ATP depletion, release of apoptotic factors and eventually cell death (Mewton et al., 2015; Ndrepepa, 2015). In this regard, the opening of MPTP constitutes a critical mediator of reperfusion injury, hence its inhibition can provide significant protection for cardiomyocyte from damage (Hausenloy et al., 2014).

Cyclosporine is a well-known immunosuppressant agent which also exhibit an effective and specific inhibitory properties against the MPTP (Waldmeier et al., 2002; Mewton et al., 2015). After discovering that MPTP opening only occurs in the first few minutes of reperfusion (Griffiths and Halestrap, 1995) has limited the time-window for using MPTP inhibitors as the therapeutic strategy to target myocardial reperfusion injury. Based on numerous experimental studies, it is believed that Cyclosporine administered at the time of reperfusion can lower lethal reperfusion injury and myocardial infarct size in patients with STEMI (Hausenloy et al., 2014; Cung et al., 2015; Mewton et al., 2015). Many trials have been done in order to assess the efficacy and safety of Cyclosporine injected immediately before reperfusion therapy in AMI patients (Piot et al., 2008; Ghaffari et al., 2013; Chiari et al., 2014; Hausenloy et al., 2014; Cung et al., 2015). Cyclosporine has been a promising therapeutic intervention at reducing reperfusion injury which will subsequently reduce the total cost of post-reperfusion injury management. However, to date there has been no review done to systematically compile all the evidences from all trials into one accessible and usable document. There is also no strong consensus regarding the use of Cyclosporine in patients with AMI. The current systematic review, therefore, aims to summarize and scrutinize the level of evidence on the safety and efficacy use of Cyclosporine in patients with AMI, in order to improve access to the evidence and the decision making.

## Materials and methods

A systematic review and meta-analysis of randomized controlled trials (RCT) was performed from January 2000 to August 2017. Studies selected for our systematic review and meta-analysis were based on criteria as follow:

### Search strategy

All articles published in English that were published in PubMed, Embase, and CINAHL databases from January 2000 to August 2017 were searched using the search terms; The following words were combined with “cyclosporin”, using the Boolean operators (“AND” & “OR”): “AMI”, “MI”, “cardiovascular stroke”, “myocardial infarct”, “PCI”, “Percutaneous coronary intervention”, “infarct size”, “ischaemia”, “myocardial stunning”, “ciclosporin”, “Cyclosporine-Neoral”, “Cya-NOF”, “Cyclosporine A”, “Neoral”, “OL-27-400”, “Sandimmun”, “Sandimmun Neoral”, “effectiveness”,“treatment outcomes”, “reperfusion injury”, “clinical effectiveness”, “clinical efficacy”, “rehabilitation outcome”, “treatment effectiveness”, and “treatment efficacy”.

We included all RCT with a control group receiving either placebo, or no intervention, and were published within the timeline and databases mentioned above. Other study designs i.e., non-RCTs, reviews, letters, case studies, conference papers, animal studies, reports or editorial, non-English literatures, report not found in print or online domain, or qualitative studies, were excluded.

### Search methods for identifications of studies

Potential studies were identified by using following keywords or subject heading: (1)AMI: “AMI” OR “MI” OR “cardiovascular stroke” OR “myocardial infact” OR “myocardial stunning”; (2) Cyclosporine: “Cyclosporine” OR “Ciclosporin” OR “Cyclosporine-Neoral” OR “Cya-NOF” OR “Cyclosporine A” OR “Cyclosporine A” OR “Neoral” OR “OL-27-400” OR “Sandimmun” OR “Sandimmun Neoral” OR “Sandimmune”; (3) Efficacy: “effectiveness” OR “treatment outcomes” OR “clinical effectiveness” OR “clinical efficacy” OR “patient-relavant outcome” OR “rehabilitation outcome” OR “treatment effectiveness” OR “treatment efficacy”; (4) Safety: “Safety” OR “Patient Harm” OR “Patient safety” OR “ADR” OR “adverse drug” OR “adverse drug reaction” OR “side effect.”

Two investigators then independently screened the identified studies; Data extraction and risk assessment of the studies were also done by all investigators independently. The Cochrane Risk of Bias Assessment tool for RCT was used to assess the quality of all included studies (Higgins et al., 2011). All conflicts emerged during quality assessment were resolved with consensus.

### Inclusion and exclusion criteria:

#### Type of participants

We included studies for original research articles if the participants were adults (aged 18 years and older) presenting with AMI. Studies that involved the participants without AMI, and children were excluded from this overview.

#### Type of interventions

We selected all studies that used Cyclosporine as their intervention regardless of dosages, formulations, and administration routes used.

#### Types of outcomes

The data was searched for outcomes in the following categories; (i) the efficacy of Cyclosporine administered in patients with AMI measured by: the left ventricular ejection fraction (LVEF), mortality and rehospitalisation secondary to worsening heart failure, recurrent MI, heart failure occurrence and infarct size (determined by serum biomarkers release: creatinine kinase myocardial band (CK-MB) and cardiac troponin I or T (cTnI or cTnT) or MRI/ CMR); (ii) the safety of Cyclosporine used in patients with AMI.

### Statistical analyses

We calculated mean difference (MD) between clinical studies for a comparison of LVEF percentage after administration of Cyclosporine in patients with AMI. Mean difference values smaller than 0 indicate a result that favors Cyclosporine compared to placebo. Odds ratios (ORs) were calculated for other outcomes such as mortality rate, rehospitalization, MI recurrence, and heart failure occurrence for comparison between clinical studies. Odds ratio values < 1 favor Cyclosporine over placebo. Fixed-effect model was used to calculate pooled MD and ORs. For each pooled MD and OR, we performed the equivalent z test and the results were considered statistical significant if p value < 0.05. Heterogeneity I^2^ value of more than 75% is a considerable heterogeneity, while I^2^ value below than 40% suggest that heterogeneity might not be important between studies (Ryan, 2014). Publication bias were presented graphically using a funnel plot. All analyses were conducted using Review Manager version 5.3 (Revman; The Cochrane Collaboration, Oxford, UK). Pre-specified subgroup analyses of patient population with occurrence of arrhythmic events, heart failure, mean LVEF at admission as well as mean LVEF at discharge (treatment vs. control groups) were performed.

## Results

### Study selection

**Figure 1:**
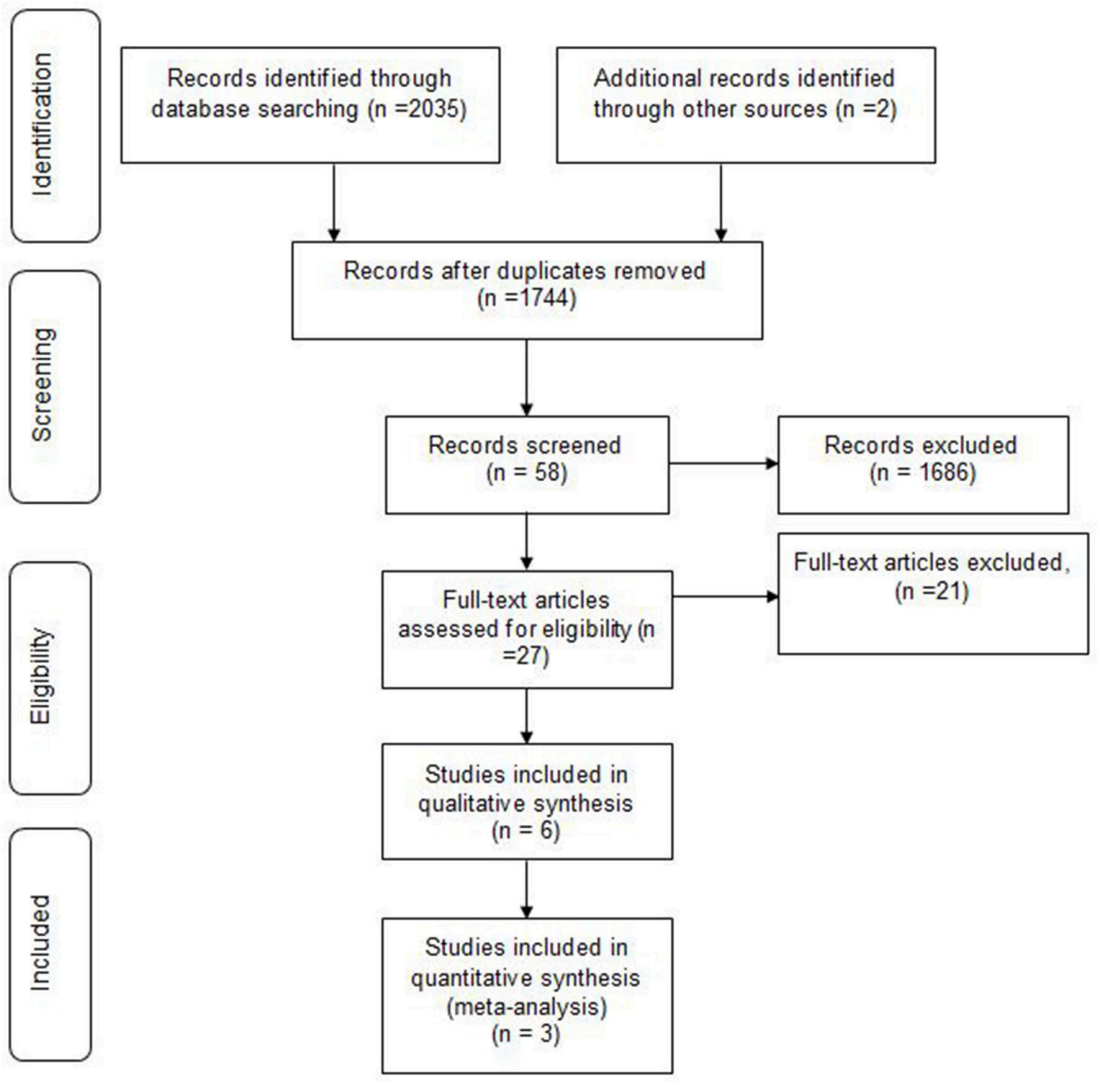
PRISMA chart.

We identified 2,037 articles, of whom 293 were duplicates. Based on inclusion and exclusion criteria, 1686 were excluded. Finally a total of 58 papers were subjected to final screening. Further screening based on title and abstract, excluded 31 papers, resulting in retrieval of 27 full papers. Of the 27 studies, 10 were excluded for not meeting the population inclusion criteria, and a further 1 1papers were excluded because they were not RCTs [letter to editor (1), case reports (4), observational study (2)s, case control (2), case series (1) and review article (1)]. Finally, only 6 randomized trials met all the inclusion criteria and were hence included in this systematic review. Data obtained during literature search process was presented using the Preferred Reporting Items for Systematic Reviews and Meta-Analyses (PRISMA) flowchart (Figure 1). No disagreement about the literature search results emerged between the three reviewers.

### Study characteristics

All six studies were parallel designed RCTs. Three were double blinded, and the other three were single blinded. Table 1 summarizes the differences in key characteristics of the six included studies.

**Table 1:** Characteristics of included studies.

**Trial & year**	**Study design and sample size**	**Clinical setting**	**Participants**	**Exclusions**	**Intervention**	**Comparator**	**Outcomes**	**Conclusion**
Cung et al., 2015	Double- blind, randomized controlled trial *N* = 970	Multicenter study sites in Belgium and Spain	Patients with anterior STEMI who were under- going PCI within 12 hours after symptom onset and who had complete occlusion of the culprit coronary artery	Patients with cardiogenic shock at admission and those with evidence of coronary collateral vessels (Rentrop score of 2 or 3 for the region at risk) on initial coronary angiography	IV bolus Cyclosporine 2.5 mg/kg before coronary recanalization N: 475	Matching IV placebo before coronary recanalization N: 495	Primary: Composite of death from any cause, worsening of heart failure during the initial hospitalization, rehospitalization for heart failure, or adverse left ventricular remodeling at 1 year (an increase of 15% or more in the LVED volume) Secondary: Changes in LVEF and LVED and LVES volumes, recurrent AMI, unstable angina, and stroke	Cyclosporine did not result in better clinical outcomes than those with placebo and did not reduce the risk of adverse left ventricular remodeling at 1 year.
Hausenloy et al., 2014	Double- blind, randomized controlled trial *N* = 78	Multicentre in UK hospitals	Adult patients referred for elective CABG surgery	Patients older than 85 years, with unstable angina, moderate or severe renal impairment, cirrhotic liver disease, immuno-compromised, taking oral glibenclamide or nicorandil	IV bolus of Cyclosporine (2.5 mg/kg) in 100 mL NaCl 0.9% over 10 min. N: 40	IV bolus of 100 mL NaCl 0.9% over 10 min N: 38	The extent of peri- operative myocardial injury (PMI) assessed by measuring serum cardiac enzymes, troponin T (cTnT) and CK-MB	Administration of a single dose of Cyclosporine prior to CABG surgery can reduce PMI in higher-risk patients with longer CPB times
Piot et al., 2008	Single- blind, randomized controlled trial *N* = 58	Multicenter at 3 study centers in France	Patients who presented with acute ST-elevation myocardial infarction	Patients with cardiac arrest, ventricular fibrillation, cardiogenic shock, stent thrombosis, previous AMI or angina within 48 hours before infarction, occlusion of the left main or left circumflex coronary artery or with evidence of coronary collaterals to the region at risk on initial coronary angiography, hypersensitivity to Cyclosporine, renal failure, liver failure, uncontrolled hypertension, immunologic dysfunction or pregnant women or women at child-bearing age not using contraception	IV bolus of 2.5 mg /kg of Cyclosporine immediately before undergoing PCI N: 30	IV normal saline N: 28	Infarct size measured by the release of creatine kinase and troponin I and MRI Cumulative incidence of major adverse events occurred within the first 48 hours after reperfusion, including death, heart failure, acute myocardial infarction, stroke, recurrent ischemia, the need for repeat revascularization, renal or hepatic insufficiency, vascular complications, and bleeding, infarct-related adverse events, including heart failure and ventricular fibrillation	Administration of Cyclosporine at the time of reperfusion was associated with a smaller infarct size as measured by the release of creatine kinase and delayed hyperenhancement on MRI. However, release of troponin I was not significantly reduced by the administration of Cyclosporine
Mewton et al., 2010	Single- blind randomized controlled trial *N* = 28	Single site in France	Patients who presented with acute ST-elevation myocardial infarction	As in Piot study	IV bolus of 2.5 mg /kg of Cyclosporine immediately before undergoing PCI N: 15	IV normal saline N: 13	LV volumes, mass, ejection fraction, myocardial wall thickness in infarcted and remote noninfarcted myocardium, and infarct size by CMR	Cyclosporine used at the moment of AMI reperfusion persistently reduces infarct size which might improve the post-infarction remodeling process and does not have a detrimental effect on LV remodeling
Ghaffari et al., 2013	Double- blind, randomized controlled trial *N* = 101	Single site in Iran	Patients with anterior STEMI	Patients with previous history of MI, primary ventricular fibrillation or cardiac arrest in the acute phase, hypersensitivity to Cyclosporine, pregnant women or in child bearing age, TLT initiated immediately before initiation of study drug, MI precipitated by a condition other than atherosclerotic coronary artery disease, systolic blood pressure < 90 mm Hg not responsive to IV fluids, any disorder that immunologic disorder, renal failure, hepatic failure, any malignancy, a positive serologic test for HIV), end-stage pulmonary disease, and contraindications for thrombolytic treatment	IV bolus of 2.5 mg /kg of Cyclosporine immediately before thrombolytic treatment N: 50	IV normal saline N: 51	In-hospital congestive heart failure, major arrhythmias (sustained ventricular arrhythmias or atrial fibrillation), or death from any cause during hospitalization and up to 6 months follow-up	The prethrombolytic administration of Cyclosporine was not associated with a reduction in the infarct size (measured by the release of cardiac enzymes) or any improvement in clinical endpoints and mortality.
Chiari et al., 2014	Single- blind randomized controlled trial *N* = 61	Single site Hospital Louis Pradel in Lyon, France	Patients scheduled for aortic valve surgery	Patients come for emergency surgery, combined aor- tic valve and coronary surgery, significant coronary stenosis, LVEF < 40%, renal insufficiency, severe hepatic dysfunction, uncontrolled hypertension, current infections, immunological disorder, taking nicorandil, sulfonylurea or rosuvastatine	IV bolus of 2.5 mg /kg of Cyclosporine immediately before undergoing PCI N: 30	IV normal saline N: 31	Primary outcome: AUC for cTnI release. Secondary outcomes: extubation time, length of stay in ICU and hospital, Simplified Acute Physiology Score and major adverse events occurring during hospitalization i.e. all- cause death, infection requiring IV antibiotic therapy and any peri- and postintervention complications.	Cyclosporine administration at the time of reperfusion protects against reperfusion injury in patients undergoing aortic valve surgery as shown by significant reduction in postoperative cTnI release.

All six studies administered the same dose of Cyclosporine (2.5 mg/ kg given as single intravenous bolus) in their treatment arms. The duration of studies varied from hospitalization stay to 1 year. The studies had different length of outcome follow up. The shortest follow up were reported in study by Piot et al. (2008),48 hours and the longest by Cung et al. (2015), 1 year.

### Quality assessment of included studies

#### The cochrane risk of bias assessment tool

**Figure 2:**
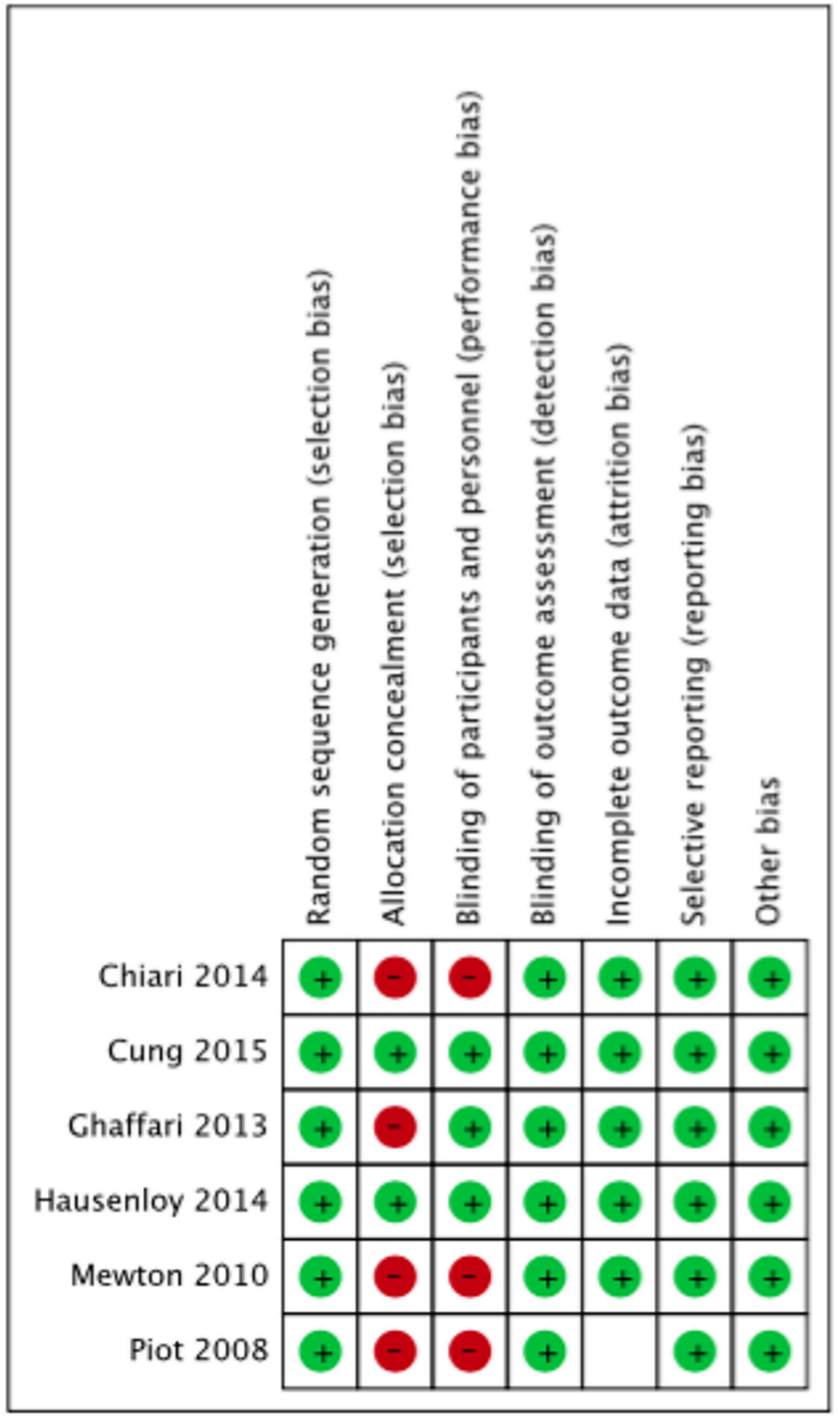
Risk of bias of included studies.

**Figure 3:**
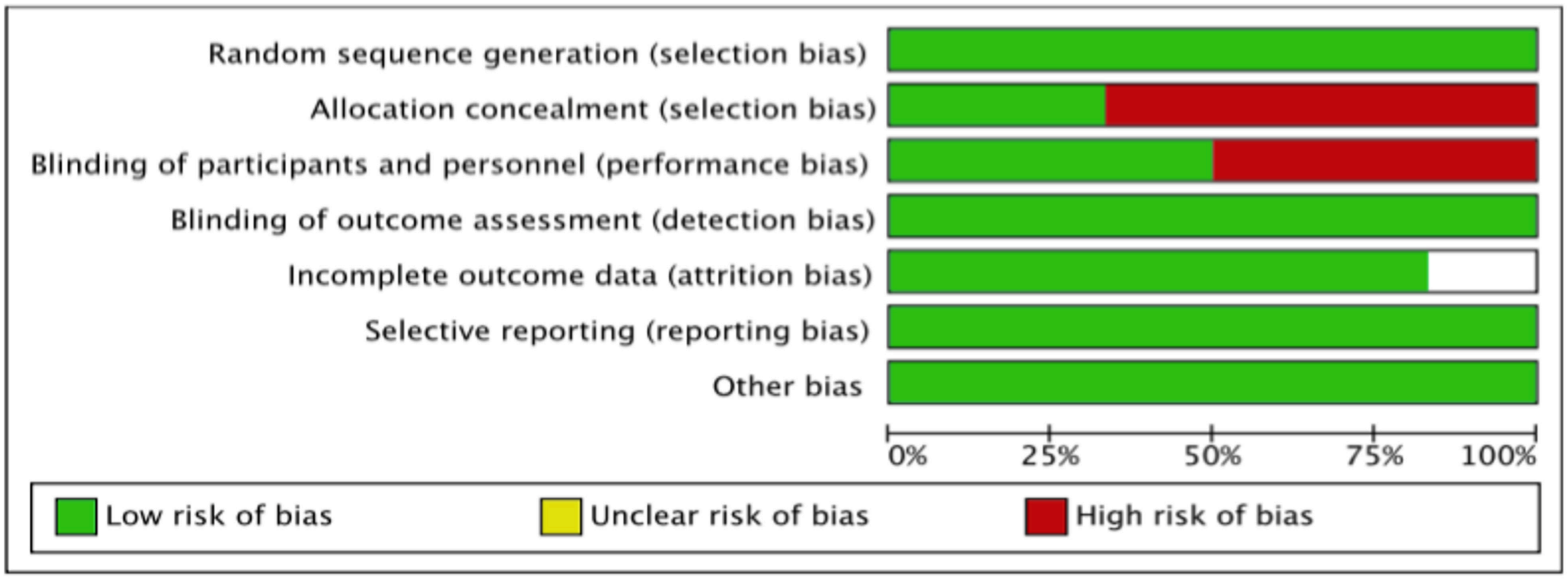
Summary risk of bias of included studies.

The summary risk of bias for included studies is shown in Figures 2, 3. Most of the studies reviewed were found to have a low risk of bias. Random sequence generation was sufficiently reported in all studies. Some of the included studies had higher risk of bias in terms of allocation concealment and blinding procedures. Four studies, i.e., Piot et al. (2008), Mewton et al. (2010), Chiari et al. (2014) and Ghaffari et al. (2013) were found to have higher risk of selection bias due to inadequate allocation concealment prior to participants' assignment. Three i.e., Piot et al. (2008), Mewton et al. (2010) and Chiari et al. (2014) out of the six studies were a single blinded studies which contributed about 50% risk to the total performance bias across studies. All of these three studies were, however, considered to have a lower risk of bias in the outcome assessment since independent expert that blinded to the assignment were recruited to analyse the data. Outcome report was adequate in all studies, except for one. Outcome data were not clearly reported by Piot et al. (2008), thus providing insufficient information to permit judgement. Of all the six studies, four studies, i.e., Piot et al. (2008), Mewton et al. (2010), Cung et al. (2015) and Chiari et al. (2014) were supported financially by the French government and one study, i.e., Hausenloy et al. (2014) was funded by then British Heart Foundation. However, all these studies were judged as low risk of other biases since sponsored parties had no conflict of interest towards the study's drug.

#### Outcome of the included studies

The description of outcomes for each study is illustrated in Table 2. In the study done by Cung et al. (2015), there was no significant difference between groups in term of level of total creatinine kinase and Electrocardiographic Data (LVEF, LVED, or LVES) at any time point (i.e., at baseline, after, at discharge, or at 1 year). The all-cause mortality at 1 year was reported to be 7.1% in the Cyclosporine group and 6.6% in the control group. The rate of initial worsening of heart failure or rehospitalization for heart failure at 1 year was similar in the Cyclosporine group and the control group (22.8 and 22.7% respectively). Adverse left ventricular remodeling occurred in 42.8% of the patients in the Cyclosporine group and in 40.7% of those in the control group. The combined incidence of death, heart failure worsening, and rehospitalization for heart failure at 1 year was similar in both groups. The rate of all other secondary clinical outcomes, including cardiogenic shock, recurrent MI, unstable angina, stroke, and acute renal failure, were similar in the two groups at 1 year.

**Table 2:** Description of outcomes included in systematic review.

**Author (year)**	**Cung et al.**, **2015**		**Hausenloy et al.**, **2014**		**Piot et al.**, **2008**		**Mewton et al.**, **2010**		**Ghaffari et al.**, **2013**		**Chiari et al.**, **2014**	
Sample size (n) Cyclosporine vs. Control	970		78		58		28		101		61	
	475	495	40	38	30	28	15	13	50	51	30	31
Length of outcome follow up	1 year		72 h		48 h and 3 months		5 days and 6 months		6 months		During hospitalization	
**STUDY PARAMETER**												
Death	28	26	–	–	–	–	–	–	9	10	1	–
Percentage (%)	7.1	6.0	–	–	–	–	–	–	18.0	19.6	3.3	–
Odds ratio (95% CI)	0.76 (0.63–1.90) *p* = 0.76								*p* = 0.99		Not mentioned	
Rehospitalisation	42	41	–	–	1	3	–	–	–	–	–	–
Percentage (%)	10.6	10.4			3.3	10.7			–	–	–	–
Odds ratio (95% CI) *P-*value	1.03 (0.65–1.63) *p* = 0.89				*p* = 0.28				
Recurrent MI	9	15	–	–	1	0	–	–	9	12	3	4
Percentage (%)	2.5	3.8			3.3				18.0	23.5	10.0	12.9
Odds ratio (95% CI) *P*-value	0.59 (0.26–1.37) *p* = 0.22				Not mentioned				*p* = 0.83		Not mentioned	
Heart Failure	62	67	–	–	1	6	–	–	18	19	4	5
Percentage (%)	15.7	16.9			3.3	21.4			36.0	38.3	13.3	16.1
Odds ratio (95% CI) *P*-value	0.92 (0.63–1.34) *p* = 0.05				Not mentioned				*p* = 0.83		Not mentioned	

Postoperative outcome measures showed that there were no significant differences in the length of hospital stay (*p* = 0.53), length of stay in the postoperative critical care unit (*p* = 0.53) and inotrope use. The balloon pump also was not needed for inotropic support in any of the patients postoperatively. Two patients (6%) in the Cyclosporine group and none of the patients in the control group presented with a postoperative wound infection.

Measurement of the area of delayed hyperenhancement (i.e., infarcted tissue), using cardiac magnetic resonance imaging (MRI) in a subgroup of patients, which was assessed on day 5 after infarction, showed that Cyclosporine significantly reduced the absolute mass of the area of hyperenhancement for about 20% as compared with the control group (*p* = 0.04) corresponding to 26 and 36% reductions in AUCs for creatinine kinase and troponin I release, respectively.

For other end points observed during the first 48 hours after reperfusion, seven adverse clinical events were recorded in the control group (one episode of ventricular fibrillation and six episodes of heart failure) as compared with three adverse clinical events in the Cyclosporine group (one episode of ventricular fibrillation, one episode of heart failure, and one episode of recurrent ischemia). When only infarct related events were considered (i.e., ventricular fibrillation and heart failure), seven events were observed in the control group vs. two in the Cyclosporine group (*P* = 0.05).

There were no other adverse events during the interval from 48 hours to 3 months. However, 3 months after infarction, three patients in the control group and one in the Cyclosporine group required rehospitalization for heart failure. These four patients were among those who had heart failure within the first 2 days after AMI. The mean LVEF at 3 months, as measured by echocardiography, was 47 ± 3% in the control group and 50 ± 2% in the Cyclosporine group.

There was a significant reduction of LVESV at 5 days and 6 months after infarction in the Cyclosporine group compared with the control group, but no significant difference was seen in LVEF as well as the left ventricular end-diastolic volume (LVEDV) between the 2 groups. Results on LV regional wall thickness showed no significant difference between the 2 groups in either the global LV mass or regional wall thickness of the remote non-infarcted myocardium or infarcted myocardium of patients, at 5 days and 6 months.

In the study by Ghaffari et al. (2013), the endpoint data for the composite in-hospital and 6-month outcomes showed that there was no significant difference with respect to major arrhythmic events [9 (18%) vs. 12 (23.5%), *P* = 0.80], occurrence of heart failure [18 (36%) vs. 19 (38.3%), *P* = 0.83], LVEF at admission [34.7 ± 9.9% vs. 33.5 ± 8.1%, *P* = 0.50] and discharge [37.7 ± 10% vs. 36.1 ± 8.2%, *P* = 0.43], and TLT-related complications [12 (24%) vs. 12 (23.5%), *P* = 1]. In-hospital [4 (8%) vs. 6 (11.8%), *P* = 0.74] and 6-month [9 (18%) vs. 10 (19.6%), *P* = 0.99] mortality rates were similar between groups.

Repeated analysis was done in subgroups of patients and again, there was no significant difference in occurrence of arrhythmic events [3 (17.6%) vs. 4 (25%); *P* = 0.69], heart failure [4 (24%) vs. 5 (31.3%); *P* = 0.71], or mean LVEF at admission (30.7 ± 9.6% vs. 34.0 ± 9.4%; *P* = 0.32), and mean LVEF at discharge (30.4 ± 8.1% vs. 33.0 ± 9.4%; *P* = 0.40) between treatment vs. control groups. Two patients in each group died at hospital and during the follow-up period (*P* = 1), among this sub-group of patients. Therefore, the pre-thrombolytic administration of Cyclosporine was not associated with a reduction in the infarct size or any improvements in clinical outcomes.

In Chiari et al.'s study (2014), a significant 35% reduction of AUC for cardiac troponin I was observed in the Cyclosporine group compared with the control group (*p* = 0.03) and this Cyclosporine effect on cTnI release remained significant after adjustment for aortic cross-clamping duration in each group (*p* = 0.01) and after further adjustment on age, sex, and LV mass index (*p* = 0.02).

For secondary outcome, it had shown that none of the treated patients had significant side effects during or after the administration of Cyclosporine indicating that Cyclosporine was well tolerated. In the postoperative period, the severity score of patients in the ICU, as assessed by the Simplified Acute Physiology Score, was comparable in both groups. Extubation time and ICU or hospital length of stay was found to be the same between the groups. The LV function, assessed by transthoracic echocardiography at hospital discharge, also did not display any differences between groups. Adverse events occurred similarly in the two groups, these were: two sepsis cases in each group with bronchitis and antibiotherapy, two pneumothorax cases in the control group, one case in Cyclosporine group of sternal instability with reoperation necessity, two cases in control group and one case in Cyclosporine group of postoperative atrioventricular block and 1 death in Cyclosporine due to complete atrioventricular block together with a temporary external pacing dysfunction. Overall, there were no significant differences between groups in the combined adverse event rate (odds ratio, 0.64; 95% CI, 0.16 to 2.55; *P* = 0.52).

### Synthesis of results

#### LVEF

**Figure 4:**
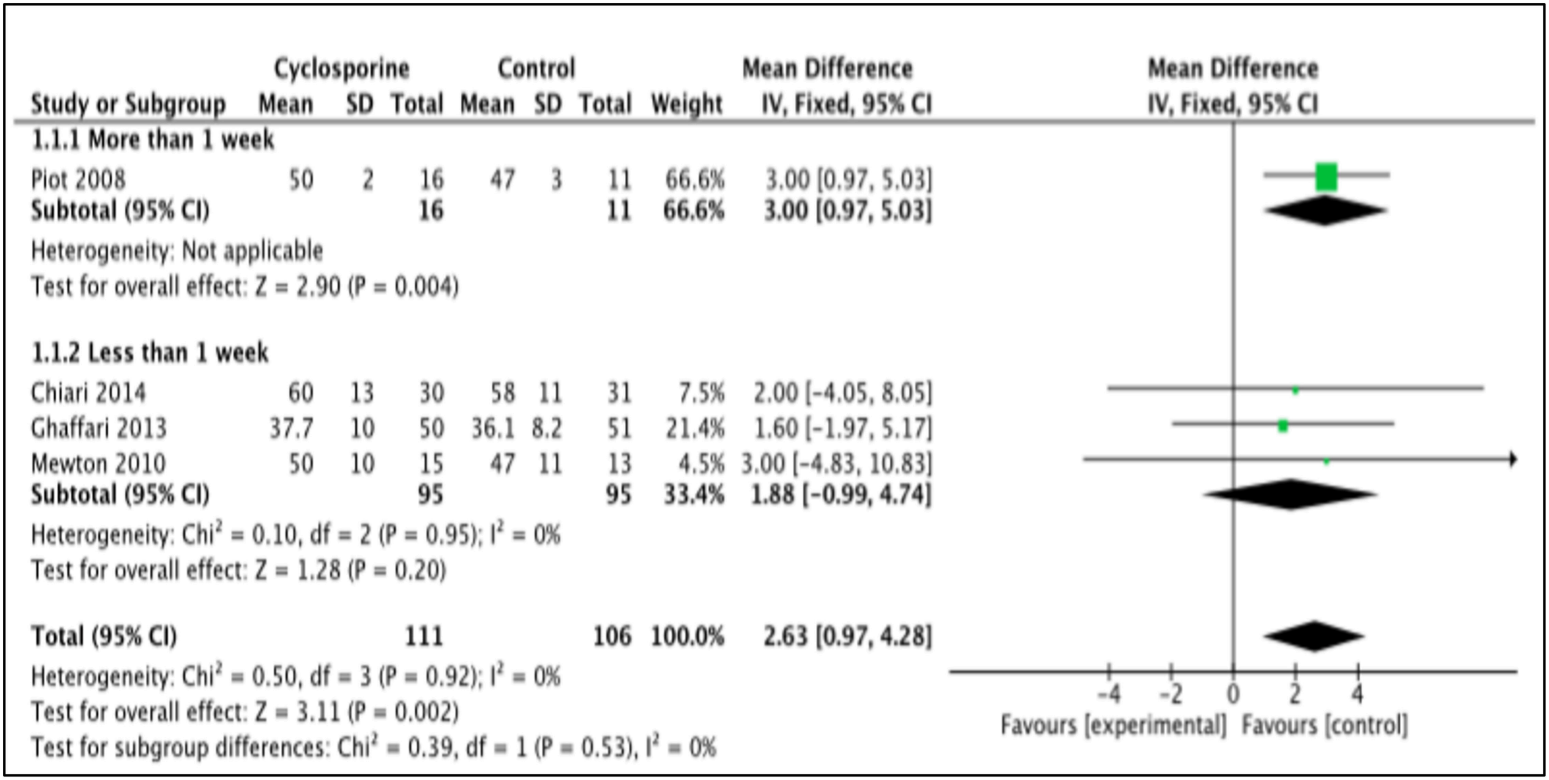
Forest plot of cyclosporine vs. placebo at improving LVEF.

**Figure 5:**
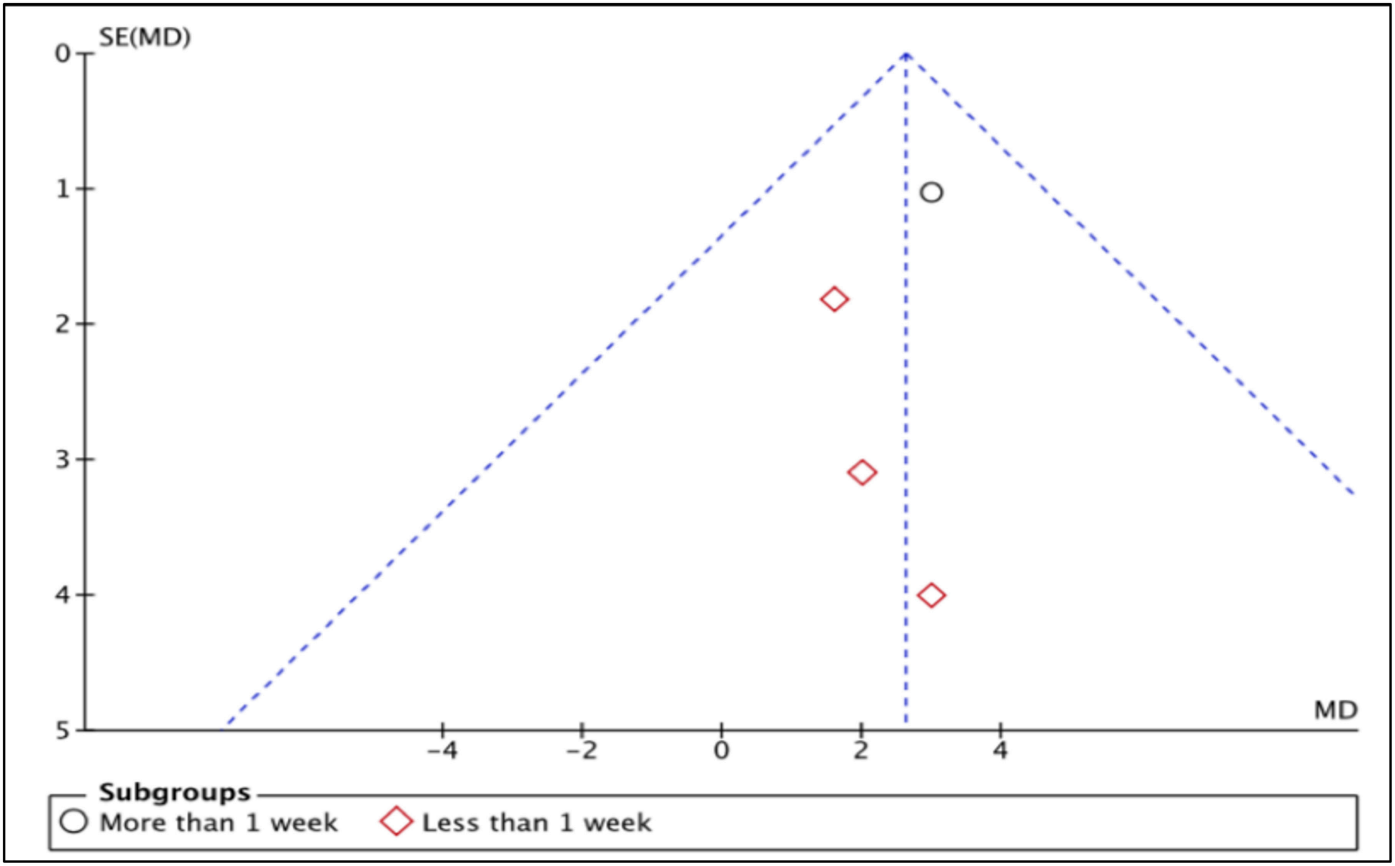
Funnel plot for the risk of publication bias of outcome LVEF.

LVEF is the measurement of how much blood is being pumped out from the left ventricle of the heart during each contraction (Cohn et al., 1974). Four studies, i.e., Piot et al. (2008), Chiari et al. (2014), Ghaffari et al. (2013), Mewton et al. (2010) reported the outcome of LVEF in percentage (%) unit. LVEF in Piot et al. was measured 1 week after intervention with Cyclosporine, while the rest measured and reported LVEF in < 1 week after Cyclosporine administration (Heterogeneity between two subgroups, *I*^2^ = 0%, *P* = 0.92). Analysis between studies showed a result that favors the control group. Results of the subgroup analysis of LVEF measured < 1 week after Cyclosporine administration showed no significant difference between Cyclosporine and placebo, suggesting no benefit of Cyclosporine at improving LVEF when given to MI patients (MD 1.88; 95% Cl −0.99 to 4.74, 3 studies, 190 patients, *I*^2^ = 0%; Figure 4). There was also no evidence of publication bias based on funnel plot inspection (Figure 5).

#### Death

**Figure 6:**
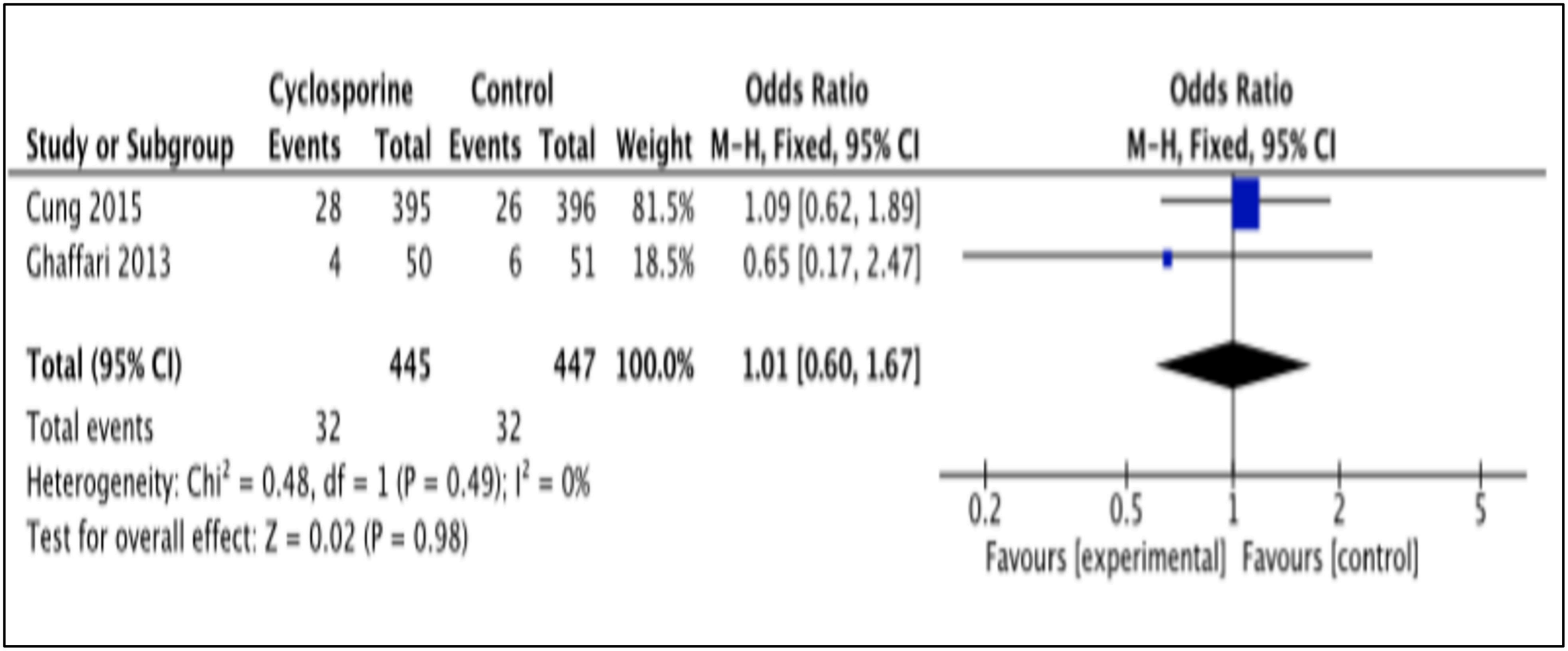
Forest plot of cyclosporine vs. placebo in preventing death.

Death occurrence after intervention with Cyclosporine and placebo was reported by 2 studies, i.e., Cung et al. (2015), Ghaffari et al. (2013). The total number of death was measured for a duration of 1 year after Cyclosporine administration by Cung et al. (2015), while Ghaffari et al. (2013) measured it for a duration of 6 months (Heterogeneity between two study is *I*^2^ = 0%, *P* = 0.49). Data in the forest plot shows the number of dead patients after 1 year and 6 month of Cyclosporine and placebo administration (Figure 6). There was no difference between the Cyclosporine and placebo groups, in preventing death (OR 1.01; 95% Cl 0.60 to 1.67, 2 studies, 892 patients, *P* = 0.98).

#### Rehospitalization

**Figure 7:**
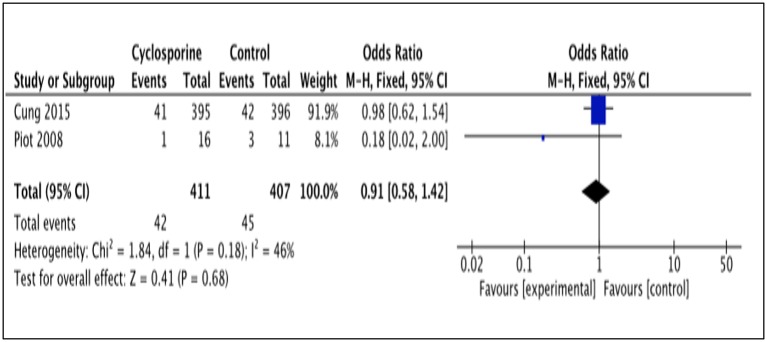
Forest plot of cyclosporine vs. placebo in preventing rehospitalisation.

Two studies (Piot et al., 2008; Cung et al., 2015) reported patients' rehospitalization after intervention with Cyclosporine and placebo. The number of patients rehospitalized in Piot et al. (2008) was reported after 3 months of intervention while Cung et al. (2015) reported it for a duration of 1 year after intervention (Heterogeneity between the two group in this analysis I^2^ = 46%, P = 0.18). This analysis showed no significant difference between Cyclosporine and placebo in preventing rehospitalisation. (OR 0.91; 95% Cl 0.58 to 1.42, 2 studies, 818 patients, *P* = 0.68; Figure 7).

#### Recurrence of MI

**Figure 8:**
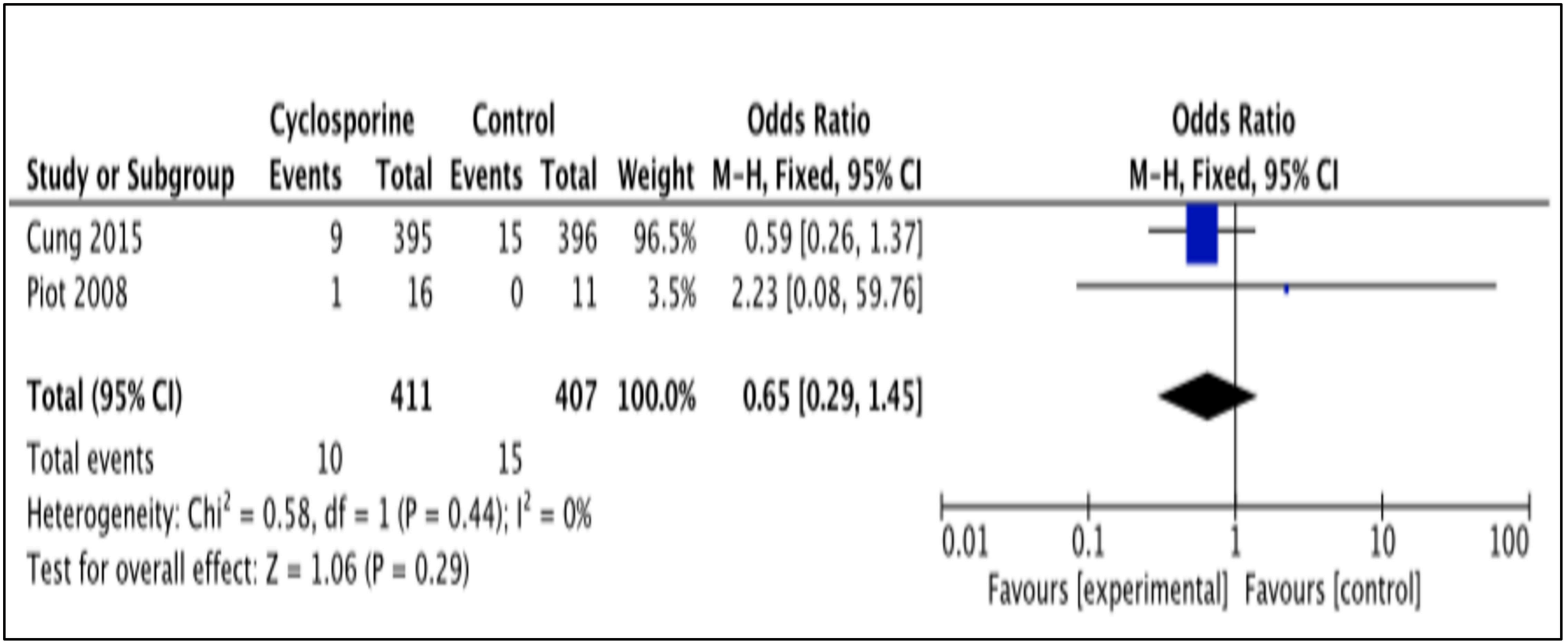
Forest plot of cyclosporine vs. placebo in preventing recurrent MI.

The number of patients who experienced recurrent MI was reported by 2 studies (Piot et al., 2008; Cung et al., 2015). The number of MI recurrence in Cung et al. (2015) was reported within 1 year after intervention while Piot et al. (2008) reported it within 48 hours after intervention (Heterogeneity between two studies is *I*^2^ = 0%, *P* = 0.44). As illustrated by the results favor Cyclosporine (Figure 8). Our analysis, however, shows no significant difference in MI recurrence prevention between Cyclosporine and placebo, either within 48 hours or up to 1 years after intervention. (OR 0.65; 95% Cl 0.29 to 1.45, 2 studies, 818 patients, *P* = 0.29).

#### Heart failure occurrence

**Figure 9:**
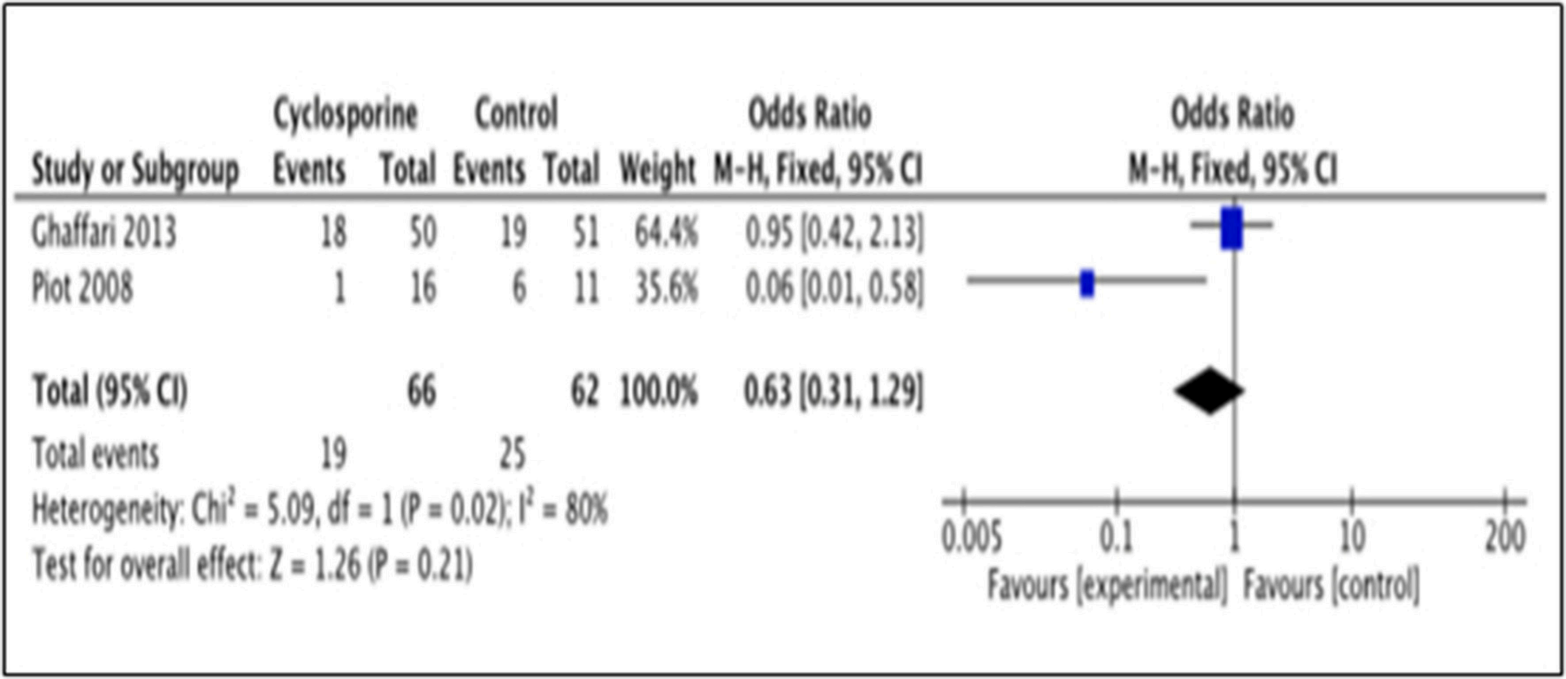
Forest plot of cyclosporine vs. placebo in preventing heart failure.

This outcome was reported by 2 studies i.e., Piot et al. (2008) and Ghaffari et al. (2013). The number of patients with heart failure occurrence in Piot et al. (2008) was measured within 48 hours after intervention while Ghaffari et al. (2013) measured it up to 6 months after intervention. Our analysis suggested no significance benefit of Cyclosporine for heart failure prevention, as compared to placebo (OR 0.63; 95% Cl 0.31 to 1.29, 2 studies, 128 patients, *P* = 0.21) (Figure 9). The large heterogeneity between the two studies (*I*^2^ = 80%, *P* = 0.02) caused a large difference in the reported prevalence of heart failure occurrence between duration of 48 h and 6 months after intervention with Cyclosporine and placebo.

## Discussion

Six randomized trials, involving a total of 1,235 patients, were included in our systematic review and meta-analysis. Four studies demonstrated that Cyclosporine might have beneficial effect to AMI patients whereas the other two studies concluded that Cyclosporine does not result in better clinical outcomes than placebo. Meta-analysis was only suitable for five outcomes: LVEF, mortality, rehospitalization, MI recurrence and heart failure occurrence. Our findings showed no significant benefit of Cyclosporine compared to placebo at improving all these outcomes. Nonetheless, not all the included studies could be pooled for the meta-analysis of these five outcomes; only four studies could be pooled for LVEF outcomes and only two studies could be included in the meta-analysis of each of the other four outcomes. The funnel plot, to detect publication bias, was therefore only done for one outcome i.e., LVEF, since visual analysis of symmetry is difficult when there are only two studies for the outcome.

The outcomes were also measured at different time points in each of the included studies, resulting in considerable heterogeneity between studies. For example, heart failure occurrence was measured within 48 h after Cyclosporine administration by Piot et al. (2008), while Ghaffari et al. (2013), measured and reported this outcome within 6 months. This variety yielded in large heterogeneity between these two studies (*I*^2^ = 80%, *P* = 0.02). Another example where heterogeneity was significant was for the rehospitalization outcome. Piot et al. (2008) reported the number of rehospitalization after 3 months of intervention while Cung et al. (2015) reported it for a duration up to 1 year contributing to moderate heterogeneity between these two studies (*I*^2^ = 46%, *P* = 0.18).

In our meta-analysis, Cyclosporine administration showed no significant difference than those of placebo, at improving LVEF when given to MI patients, without evidence of heterogeneity and publication bias. This finding is in line with that of the meta-analysis done by Song et al. (2015) to evaluate the effect of Cyclosporine on reperfusion injury in patients with AMI. Their also concluded that there was no significant improvement in term of LVEF in Cyclosporine group compared to control group, with no evidence of heterogeneity (Song et al., 2015). In contrast, in one experimental study, Cyclosporine was found to be associated with better LV function when compared with control group in animal model of ischaemia and reperfusion (Zalewski et al., 2015).

The use of Cyclosporine for infarct size reduction always conflicted in evidence. In a meta-analysis that resulted in 20 20 articles on animal models Cyclosporine was demonstrated to reduce infarct size only in two third of studies, with the effect of heterogeneous (Lim et al., 2012). In one randomized trial done on humans also showed that intravenous Cyclosporine reduced the infarct size as measured by CK-MB and CMR. However, when infarct size was measured by troponin I, no change was observed (McAlindon et al., 2014).

Some studies could not be included in the meta-analyse due to insufficient reported data. These include the following: angiography finding and major arrhythmic event by Ghaffari et al. (2013); LV volume and LV wall thickness by Mewton et al. (2010); adverse left ventricular modeling, cardiac shock, stroke and major bleeding by Cung et al. (2015); and ventricular fibrillation by Piot et al. (2008). Another outcome that could not be included was the effect of Cyclosporine in reducing infarct size reportedby Piot et al. (2008) and Mewton et al. (2010) who were used different parameters. Specifically, Piot et al. (2008) reported median ± interquartile range, whereas Mewton et al. (2010) reported mean ± SD.However, both studies found significant shrinkage of the infarcted tissue area in the Cyclosporine group as compared with the control group [*p* = 0.04 by Piot et al. (2008); *p* = 0.03 at 5 days and *p* = 0.04 at 6 months by Mewton et al. (2010)].

Cyclosporine is an immunosuppressive agent that is often associated with adverse events after chronic use, such as nephrotoxicity, hypertension, hyperlipidaemia, neurotoxicity, hepatotoxicity, anorexia, nausea, vomiting, paraesthesia, hypertrichosis, gingival hyperplasia, and tremor (Hausenloy et al., 2012). In this review, we were able to find four studies [Cung et al. (2015), Piot et al. (2008), Chiari et al. (2014), and Hausenloy et al. (2014)] that reported the observed adverse reactions in their studies. Considering that Cyclosporine was administered as a single bolus of Cyclosporine in all studies, it would be expected to have resulted in minimal adverse effects. Indeed, none of the treated patients had significant side effects during or after the administration of Cyclosporine as observed in Chiari et al. (2014) and no evidence of acute renal or hepatic injury, hypertension or other short-term adverse effects was observed by Piot et al. (2008). Similar findings were reported by Cung et al. (2015), whereby no adverse effects were observed on renal function, white-cell count, or blood glucose level. Hausenloy et al. (2014) also reported that the administration of Cyclosporine as a single intravenous bolus was found to be safe, with no related adverse effects and with no difference in peri-operative serum markers for renal and liver function. Furthermore, those review studies also concluded that there were no significant differences between Cyclosporine group and control group in the combined adverse event rate for major adverse events reported such as sepsis, sternal instability, postoperative atrioventricular block, vascular complication and bleeding. Thus, overall, it can be concluded that administration of Cyclosporine is well tolerated and adverse events occurred similarly in the two groups as reported by various experimental on animal studies.

## Conclusion

The cumulative evidence from our systematic review and meta-analysis reveals that Cyclosporine given to patients with AMI does not have better clinical outcome compared with placebo at improving LVEF, mortality, rehospitalisation, recurrent MI and heart failure occurrence. This study, however, verifies that Cyclosporine is well tolerated when administered in patients with AMI. The interpretation of these findings should take into consideration, the selection and performance bias across all studies. In addition to that, the low quality of included studies and the present of heterogeneity between studies may also have contributed to inaccurate results. More studies are needed in the future to examine the effectiveness of Cyclosporine in AMI, which should include the non-RCT articles and articles from other databases as well. Although cyclosporine holds limited promise as a cardioprotective agent for reducing reperfusion injury in STEMI, we should still uphold the potential of MPTP inhibitors in improving the clinical outcomes of reperfusion therapy. Perhaps, there is a need for the search of a novel cardioprotective agent or better strategies to limit reperfusion injury.

## Author contributions

FR, SA, WM, SS, TMK, and LT-HT: Perform the initial search and manuscript writing; data analysis was performed by FR, SA, WM, DB-CW, TMK, K-GC, C-FN, LM, L-HL, and B-HG: Have given critical comment on the final version. Manuscript was finalized by TMK, C-FN, LM, L-HL, and B-HG.

### Conflict of interest statement

The authors declare that the research was conducted in the absence of any commercial or financial relationships that could be construed as a potential conflict of interest.
